# It’s a Process: Reactions to HIV Diagnosis and Engagement in HIV Care among High-Risk Heterosexuals

**DOI:** 10.3389/fpubh.2017.00100

**Published:** 2017-05-10

**Authors:** Alexandra H. Kutnick, Marya Viorst Gwadz, Charles M. Cleland, Noelle R. Leonard, Robert Freeman, Amanda S. Ritchie, Talaya McCright-Gill, Kathy Ha, Belkis Y. Martinez, The BCAP Collaborative Research Team, Angela Banfield

**Affiliations:** ^1^Center for Drug Use and HIV Research, Rory Meyers College of Nursing, New York University, New York, NY, United States

**Keywords:** mixed methods, HIV, diagnosis, high-risk heterosexuals, HIV care continuum, HIV care engagement, antiretroviral initiation, acceptance

## Abstract

After HIV diagnosis, heterosexuals in high-poverty urban areas evidence delays in linkage to care and antiretroviral therapy initiation compared to other groups. Yet barriers to/facilitators of HIV care among these high-risk heterosexuals are understudied. Under the theory of triadic influence, putative barriers to HIV care engagement include individual/attitudinal-level (e.g., fear, medical distrust), social-level (e.g., stigma), and structural-level influences (e.g., poor access). Participants were African-American/Black and Hispanic adults found newly diagnosed with HIV (*N* = 25) as part of a community-based HIV testing study with heterosexuals in a high-poverty, high-HIV-incidence urban area. A sequential explanatory mixed-methods design was used. We described linkage to HIV care and clinical outcomes [CD4 counts, viral load (VL) levels] over 1 year, and then addressed qualitative research questions about the experience of receiving a new HIV diagnosis, its effects on timely engagement in HIV care, and other barriers and facilitators. Participants were assessed five times, receiving a structured interview battery, laboratory tests, data extraction from the medical record, a post-test counseling session, and in-person/phone contacts to foster linkage to care. Participants were randomly selected for qualitative interviews (*N* = 15/25) that were recorded and transcribed, then analyzed using systematic content analysis. Participants were 50 years old, on average (SD = 7.2 years), mostly male (80%), primarily African-American/Black (88%), and low socioeconomic status. At the first follow-up, rates of engagement in care were high (78%), but viral suppression was modest (39%). Rates improved by the final follow-up (96% engaged, 62% virally suppressed). Two-thirds (69%) were adequately retained in care over 1 year. Qualitative results revealed multi-faceted responses to receiving an HIV diagnosis. Problems accepting and internalizing one’s HIV status were common. Reaching acceptance of one’s HIV-infected status was frequently a protracted and circuitous process, but acceptance is vital for engagement in HIV care. Fear of stigma and loss of important relationships were potent barriers to acceptance. Thus, partially as a result of difficulties accepting HIV status, delays in achieving an undetectable VL are common in this population, with serious potential negative consequences for individual and public health. Interventions to foster acceptance of HIV status are needed.

## Introduction

To eliminate HIV transmission in the United States, persons living with HIV (PLWH) must be aware of their diagnoses, engage in regular HIV medical care, initiate antiretroviral therapy (ART), and adhere well to ART, in order to achieve viral suppression (1, 2). Although rates of engagement are improving (3), serious gaps are evident at every stage along the HIV care continuum (4). The Centers for Disease Control and Prevention (CDC) estimate that of the 1.2 million Americans living with HIV, 60% are not appropriately retained in HIV care; 63% are not taking ART; and 70% have detectable HIV viral load (VL) (5). Moreover, racial/ethnic disparities persist in HIV health outcomes. African-American/Black and Hispanic populations, who are disproportionately located in the lower socioeconomic strata, evidence higher rates of undiagnosed HIV than Whites (6). Moreover, among those living with HIV, African-American/Black and Hispanic persons experience greater morbidity and earlier mortality than their White peers (7, 8).

The present study focuses on African-American/Black and Hispanic heterosexuals newly diagnosed with HIV infection as part of a community-based HIV testing study. Consistent with the National HIV Behavioral Surveillance system of the CDC, we define high-risk heterosexuals as those socially connected to urban geographical areas with elevated rates of both socioeconomic disadvantage and HIV prevalence (9). In fact, heterosexual sex is the second most common route of HIV transmission in the United States after male-to-male sexual contact, accounting for an estimated 24% of newly reported infections annually, and it is by far the main route of transmission among women (10). Nationally, HIV prevalence among high-risk heterosexuals, who are predominantly African-American/Black and Hispanic, is higher than among the underlying general heterosexual population (2.3 vs. 0.6%) (11). Of concern, late HIV diagnosis is common among high-risk heterosexuals, even compared to vulnerable risk groups such as men who have sex with men (MSM) (12). Compounding the problem of late diagnosis, timely linkage to care after HIV diagnosis, a critical aspect of the effort to eliminate HIV transmission, also tends to be delayed in this group (13, 14). For example, heterosexual men evidence lower CD4 cell counts at the time HIV care is initiated and faster progression to AIDS than men in other risk categories (15). Heterosexual women living with HIV typically show delayed entry into care, delays in ART initiation, and lower rates of ART initiation compared to men, with attendant poor outcomes (16). Yet with respect to factors that impede or promote engagement along the HIV care continuum, high-risk heterosexuals are under-studied compared to other risk groups such as MSM and persons who inject drugs (17), in part because public health researchers historically lacked an accepted definition of this vulnerable population (9).

Theoretical barriers to engagement in HIV primary care and uptake of ART among newly diagnosed high-risk heterosexuals can be conceptualized using the theory of triadic influence (18), a social/cognitive theory describing three streams of influence on health behavior: individual/attitudinal, social, and structural. Existing literature indicates that barriers to timely engagement along the HIV care continuum for African-American/Black and Hispanic high-risk heterosexuals include individual/attitudinal-level impediments such as distrust of medical settings, fear of ART, low self efficacy to manage care/ART, substance use, and depression (14, 19–22); social-level barriers including stigma and social norms unsupportive of HIV care (23); and structural-level barriers such as poor access to settings where high-quality HIV care is offered, all complicated by poverty (15, 19, 24, 25). Theoretically, these factors combine synergistically to reduce motivation to engage in HIV care and initiate ART, as well as access to HIV care settings.

Moreover, a small number of recent studies have focused on an additional individual/attitudinal-level factor among vulnerable populations—acceptance of a new HIV diagnosis. In a qualitative study of MSM and women, Baumgartner and David (26) described a three-step longitudinal process by which a new HIV diagnosis is incorporated into one’s sense of self: diagnosis, a post-diagnosis turning point (usually taking place about a year after diagnosis), and then integration. Similarly, Hult and colleagues (27) found responses to a new diagnosis vary greatly, ranging from individuals being too shocked to comprehend the information, to immediately accepting the diagnosis and being ready to face next steps, including engaging in medical care. Yet overall, little is known about individuals’ reactions to receiving a new HIV diagnosis and how these reactions might promote or impede timely engagement along the HIV care continuum, including among high-risk heterosexuals.

The present mixed-methods study focuses on high-risk heterosexuals newly diagnosed with HIV. In response to the gaps in the literature described above, we first describe patterns of linkage to HIV care and clinical outcomes (CD4 counts and VL levels) over a 1-year period, to identify both successes and gaps, using quantitative data. Then, in response to these quantitative findings, we address a set of qualitative research questions focused on how individuals experience a new HIV diagnosis and the effects of this experience and other multi-level factors on timely engagement along the HIV care continuum. Finally, we discuss how findings that emerged from the qualitative data can be used to provide a deeper understanding of the patterns of engagement in HIV care after initial diagnosis and of barriers to such engagement.

## Materials and Methods

The present study used a sequential explanatory mixed-methods design (28) with a sample of high-risk heterosexuals found newly diagnosed with HIV in a larger study, described briefly below (25). We obtained quantitative descriptive data over a 1-year time period on HIV care engagement and clinical outcomes (CD4 counts and VL levels) from structured interviews, medical record data, and/or laboratory reports. Then, in response to quantitative findings, qualitative research questions were refined, in order to explain and foster interpretation of the findings of the quantitative study component (28). The qualitative component took a descriptive, multiple case study approach (29), a method that elicits participants’ in-depth descriptions of their own views and reality, which, in turn, provides a deep understanding of their choices and actions (30). Case studies are particularly useful when contextual conditions, such as cultural, social, and structural barriers to HIV care after diagnosis (e.g., fear, stigma) are potentially relevant to the phenomenon under study (29). Finally, we integrated and interpreted the entire analysis. The study received ethical approval from the New York University School of Medicine Institutional Review Board and was registered with http://ClinicalTrials.gov (NCT01607541).

### Participants

Participants were those identified as newly diagnosed with HIV in a larger study designed to seek out high-risk heterosexuals with undiagnosed HIV. Participants were recruited using respondent-driven sampling (RDS), a peer-to-peer recruitment method, and provided with confidential HIV counseling and testing, as described in brief below and in detail elsewhere (25). The rate of newly diagnosed HIV identified in this larger study was 1% (*N* = 25), and these newly diagnosed individuals are the focus of the present study.

### Eligibility

Eligibility criteria were as follows: age 18–60 years, sexually active with at least one opposite-sex partner in the past year, African-American/Black or Hispanic race/ethnicity, resides in a defined high-risk urban geographical area with elevated rates of prevalent HIV and poverty, comprehends English or Spanish, not actively psychotic, and found newly diagnosed with HIV in the larger study’s first phase (19).

### Brief Description of the Larger Study’s First Phase

**Figure 1:**
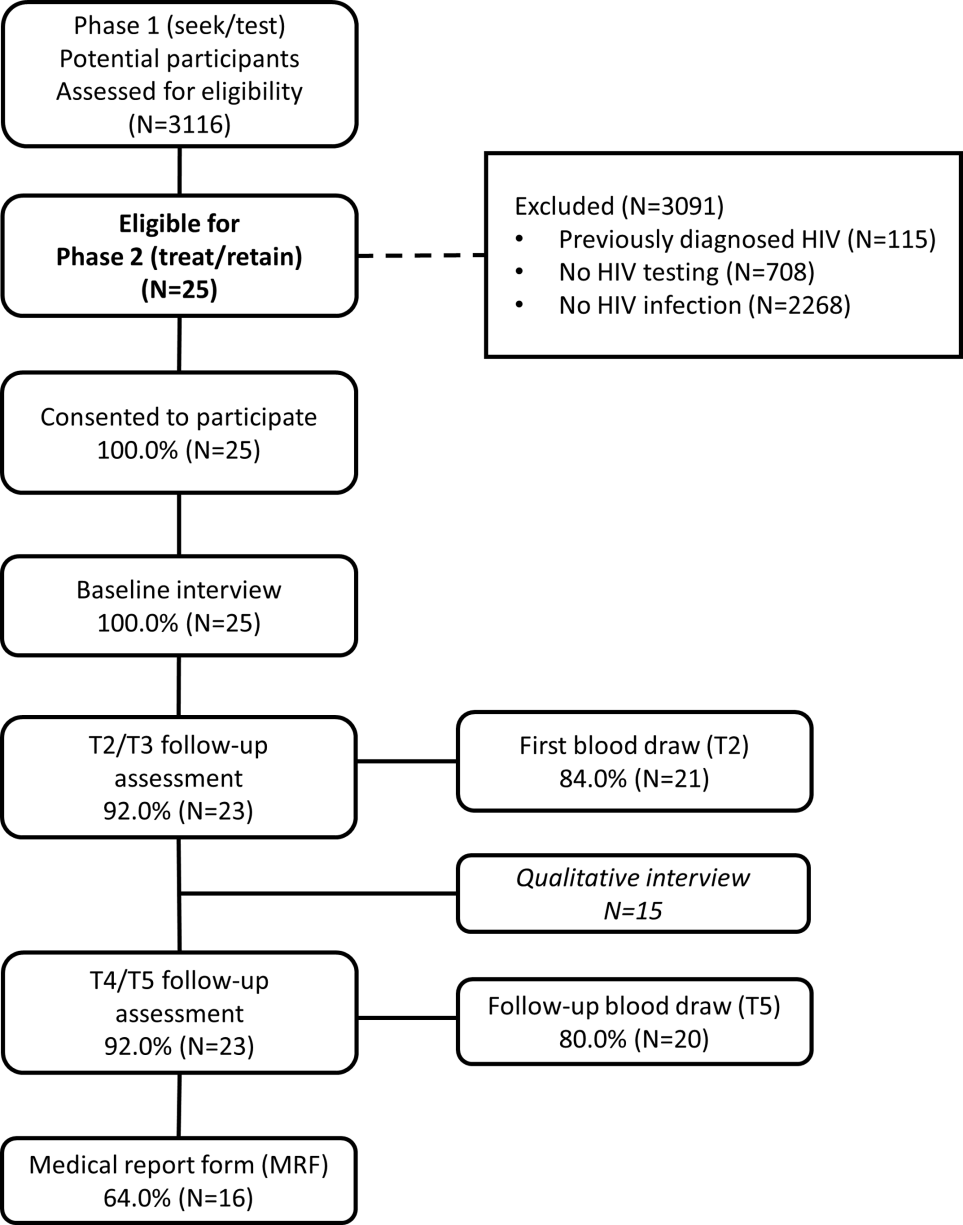
**Retention in study activities**.

In recent research, we reported on the first phase of the larger study designed to locate high-risk heterosexuals with undiagnosed HIV infection in New York City, using a “Seek, Test, Treat, and Retain” approach (31). In the past research, we examined the yield, that is, proportion of newly diagnosed HIV infection identified, of a two-arm peer-driven intervention that used RDS as its recruitment method to actively seek out high-risk heterosexuals in their communities and provide HIV counseling and testing. Further, individuals with past HIV diagnoses could be enrolled in the study and received similar activities and compensation levels, to reduce participants’ incentives to mask their HIV-positive status in order to enter the study (*N* = 115 participants with past HIV diagnosis entered the study, as shown in Figure 1) (19). Activities for those enrolling with past HIV diagnoses are not described here.

The intervention provided in both arms of this first phase was culturally based, to address barriers to HIV testing specific to African-American/Black and Hispanic individuals, and grounded in motivational interviewing, a flexible, collaborative counseling method that actively engages, focuses, and guides participants in order to elicit and strengthen high-quality, durable intrinsic motivation for behavior change (32). Because intervention activities in peer-driven intervention begin at the time of recruitment, participants recruited by peers in RDS were assigned to the intervention arm of the individual who recruited them. The two intervention arms are described in more detail elsewhere (19). In short, participants in both arms were recruited into the study by peers, engaged in a structured intervention session focused on (1) engagement into the study, (2) an orientation to study components, and (3) boosting motivation to conduct peer recruitment and HIV testing in the second session. Next participants had the opportunity to recruit peers, and then within 2 weeks presented to the study for a second session that included HIV pre-test counseling and HIV testing. This period of approximately 2–4 weeks before HIV testing was designed to give participants time to consider the upcoming HIV test experience, given they were not seeking HIV testing at the time of study enrollment, may not have considered themselves at risk for HIV compared to other risk groups such as MSM, but may nonetheless encounter HIV stigma if diagnosed. Consistent with past research on HIV testing interventions (2), we did not hypothesize differences in rates of newly diagnosed HIV between these two arms. Instead, we hypothesized in the study’s second phase, the peer-driven intervention arm, which provided patient navigation post-diagnosis compared to standard care among those newly diagnosed, as described below, would evidence greater efficacy on the outcomes, including shorter time to an HIV clinic appointment and higher rates of HIV viral suppression. However, the rate of newly diagnosed HIV identified (1%; *N* = 25; 5 intervention arm, 20 control arm) was lower than hypothesized based on past surveillance studies (9, 33)—a sample size that does not allow for a quantitative comparison of the two intervention arms. Thus in light of these findings in the larger study’s first phase, *the present study collapses the two intervention arms* for quantitative analyses and examines the cohort of newly diagnosed participants taken together. We attend to differences in study arms in the qualitative component. Participants provided signed informed consent for study activities.

### Procedures in the Present Study

#### Intervention Session and Follow-up Contact

Participants with a preliminary positive HIV rapid test result in the larger study’s first phase presented for an intervention session lasting up to 90 min. In this session, confirmatory HIV test results (from a laboratory test) and post-test counseling were provided, following the CDC and local (New York State Department of Health) guidelines for post-test counseling. These guidelines included an initial risk reduction and disclosure plan and an appointment for HIV care made for participants. Participants received compensation for the session ($30), plus local round-trip public transportation. After this session, those assigned to the intervention arm received patient navigation (3 months of intensive navigation and 3 months of maintenance navigation). Navigation is an efficacious, flexible, individualized, strengths-based approach to assist PLWH in identifying and overcoming barriers to health services (34, 35). It included in-person visits, accompaniment to health-care appointments if needed, and phone contacts. Those in the control arm received the standard of care; namely, up to four phone contacts to determine whether the health-care appointment was attended and to identify and help resolve barriers and/or encourage attendance if appropriate. The intervention and other contacts with participants in both arms were guided by motivational interviewing (36). In keeping with the motivational interviewing approach, study activities and components were designed to communicate an ethos of acceptance, non-judgment, and respect for autonomy.

#### Outcomes

Primary study outcomes for the quantitative study component included time to an HIV clinic appointment (i.e., whether linked to care within 3 months), time to initiating ART, HIV VL suppression, and retention in care among the newly diagnosed. Participants were considered retained in care if they saw an HIV care provider at least three times in the year after diagnosis, with two visits at least 90 days apart (37).

#### Assessments

Participants received a structured assessment battery at five time points: baseline in the larger study’s first phase, the time of initial diagnosis [called the Time 2 (T2) assessment], and 3 (T3), 6 (T4), and 12 months post-diagnosis (T5). The T2, T3, and T4 interviews assessed the prior quarter, and the T5 interview assessed the prior 6 months. All assessments were conducted using audio computer-assisted self-interviewing (ACASI) software and lasted approximately an hour. Participants received $30 in compensation for assessments. For parsimony in analysis, we combined the T2 and T3 assessments and T4 and T5 assessments. Retention rates were high: most completed T2 and/or T3 (92%; 23/25), and T4 and/or T5 (92%; 23/25), as shown in Figure 1.

#### Qualitative Interviews

From the 25 participants found newly diagnosed with HIV, 15 were selected at random for participation in qualitative interviews (11 male, 4 female). We conducted one-on-one, semi-structured, in-depth qualitative interviews in a confidential location at the study’s community-based field site. Interviews were conducted by experienced qualitative researchers, lasted 60–90 min and were audio-recorded and professionally transcribed verbatim. Identifying information was removed from transcripts. Interviews were conducted 8–11 months post-enrollment. Participants were compensated $30 and provided with funds for round-trip local public transportation.

#### Qualitative Interview Guide

Qualitative interviews followed a semi-structured guide that included main questions and probes linked to domains of the theory of triadic influence and known or hypothesized barriers to linkage to HIV care (e.g., stigma, fear, medical distrust, substance use, and structural barriers), and also allowed for emergent themes (e.g., delayed acceptance of diagnosis, barriers to acceptance). Questions focused on the experience of receiving the new HIV diagnosis, experiences of linkage to HIV care, barriers to and facilitators of linkage to HIV care, retention in care, and initiation of and adherence to ART.

#### Case Notes

Clinical case notes from intervention sessions, navigation, and phone contacts with participants with respect to barriers to and facilitators of linkage to care and related issues were recorded in a confidential database and included in the qualitative data set. The number of navigation contacts, both in-person and phone, ranged from 6 to 51 contacts (M = 27.2 contacts, SD = 16.3 contacts), some of them brief.

#### Biomarker Testing (VL, CD4)

Participants provided a blood specimen at T2 and T5 for testing of CD4 count and VL. Participants received $25 in compensation for blood draws. Blood specimens were processed by commercial laboratories. VL was tested using the COBAS Amplicor Ultrasensitive HIV-1 RNA PCR assay (Version 2.0), viral suppression/undetectable VL was defined as VL < 50 copies/ml. Most participants provided blood specimens at T2 (84%) and T5 (80%), as shown in Figure 1.

#### Medical Report Forms (MRFs)

At T5, a medical report form was completed by the participant’s health-care provider, extracting data on attendance at care appointments, to assess retention in care, CD4 values, and VL values over the past year. An MRF was received for 64% of participants (Figure 1). When both laboratory reports and MRF data were available, the median value was used for that participant.

### Measures

The measures used in the present study were drawn primarily from a set of harmonized instruments used for the set of Seek, Test, Treat, and Retain studies sponsored by the National Institute on Drug Abuse (NIDA) at the National Institutes of Health (38). These measures have been used in past studies with high-risk heterosexuals and similar vulnerable populations. They are described briefly below and in more detail elsewhere (22, 39). Cronbach’s alpha (α) is provided for scales where appropriate.

#### Sociodemographic and Background Characteristics

Characteristics such as age, race/ethnicity, gender, education level, insurance, housing status, employment, and financial insecurity (e.g., how often unable to pay for necessities) were measured using a structured NIDA-harmonized instrument (40).

#### Physical and Mental Health

We assessed general health on a five-point Likert-type scale [scores were collapsed to indicate whether general health was “good” or better (yes/no) (41)]. Depression over the past week was assessed with the Center for Epidemiologic Studies Depression Scale (20-items; α = 0.80) (42). A composite depression score was calculated; 16 or greater indicated presence symptoms at a clinically significant level.

#### Substance Use

Frequency of drug and alcohol use in the past month was assessed (40, 43), along with lifetime and past month history of injection drug use (41), drug problems in the past year (9 items; α = 0.91) (44), and alcohol problems in the past year (10 items; α = 0.89) (40, 45). Problematic drug and alcohol use were calculated according to established criteria (46).

#### Sexual Behavior

The National HIV Behavioral Surveillance System measure (40, 47) was used to gather data on past month sexual partners, unprotected sex, and lifetime engagement in transactional sex (i.e., exchange of sex for money, drugs, or a place to stay).

#### HIV Health Behavior

HIV health behavior, including engagement in HIV care, where care was received, initiation and continuation of ART, and whether CD4 and VL tests were received, were assessed with a measure from the HIV Cost and Services Utilization Study (HCSUS) (48).

### Analysis

#### Statistical Analysis

Descriptive statistics including counts, percentages, means, and SDs are presented in analyses. Figures showing CD4 and HIV VL over time were constructed using the ggplot2 R package (49). All analyses were conducted in the R statistical computing environment (50).

#### Qualitative Analysis

We applied a systematic content analysis approach that was both theory-driven and inductive (51, 52). Data were analyzed in the Dedoose platform (53). Case notes from navigation, phone contacts for those in the control arm, and in-depth qualitative interview transcripts were used as data sources for this analysis, creating a prospective longitudinal, qualitative data set. The research team developed a set of initial descriptive codes based on a review of transcripts, reflecting the domains of the theoretical model. Next, a trained qualitative analyst coded each interview transcript using these codes, refining and creating additional codes as necessary. At regular intervals through the coding process, a reliability check of the coding was conducted with a second qualitative analyst, who independently coded half the transcripts. Thus inter-coder reliability was established, and the existing set of codes was further refined. Discrepancies in coding were resolved by consensus. Then, we used the memo and code co-occurrence functions within Dedoose to support the analysis of relationships between and among codes and thereby develop larger themes. Next, the two qualitative analysts together identified areas of congruence and discrepancy with respect to the broader themes, exploring both manifest and latent themes (54). Then, in an iterative data analysis process, a small number of specific cases were selected to reflect the primary themes (29). Methodological rigor of the analysis was maintained through an audit trail of process and analytic memos and periodic debriefing with the larger research team, which included experts in high-risk heterosexuals and HIV care continuum issues (55).

#### Data Integration

We evaluated concordance between specific case study participants’ descriptions of their engagement in HIV care and ART and quantitative ratings derived from MRFs and T2 and T5 biomarker test results collected by the study. Thus qualitative data, case notes, and medical data were triangulated to enhance data quality (30). Findings that emerged from the qualitative data were used to provide a deeper understanding of patterns of engagement in HIV care after initial diagnosis and of barriers to such engagement, as discussed below.

## Results

### Quantitative Results

Table 1 describes participant demographics, health, substance use, and sexual behavior. Participants were 50 years old, on average (M = 49.4 years; SD = 7.2 years), mostly male (80%), and primarily non-Hispanic African-American/Black (88%). Most (72.0%) were unable to pay for basic necessities in the past year, an indication of extreme poverty. Histories of homelessness (68%) and incarceration (76%) were common. Most had health insurance (88.0%). With respect to health, 96% described their health as “good” or better. About a third had used drugs in the past month (29%), and 44% met criteria for an alcohol or drug problem in the past year. Sixteen percent of the cohort had ever injected drugs in their lifetimes. Among males, almost a third (30%) reported a non-heterosexual sexual orientation (e.g., bisexual, queer) and/or reported sex with male partners in the past. Regarding transactional sex, 48% of the sample had exchanged sex for money, drugs, or place to stay over their lifetime.

**Table 1:** **Sociodemographic and background characteristics and health factors (*N* = 25)**.

	Mean (SD)/%
Age in years	49.4 (7.2)
Male sex	80.0
African-American, not Hispanic	88.0
Latino/Hispanic	12.0
Married or in long-term relationship	52.0
No high school diploma	44.0
Current full-time or part-time work	12.0
Unable to pay for necessities in past year	72.0
Portion of income includes government benefits	52.0
Ever homeless	68.0
Currently homeless	16.0
Ever been incarcerated for >24 h	76.0
Incarcerated in the past year for >24 h	24.0
Currently has health insurance	88.0
**Health and health-related factors**	
General health “good” or better	96.0
Clinically significant symptoms of depression (Center for Epidemiologic Studies Depression Scale)	60.9
**Substance use**	
Any drug use in the past month	29.2
Drug use frequency past month (range 0–8)	0.8 (1.9)
Ever injected drugs not for a medical reason	16.0
Injected drugs in the past month	0.0
Meets AUDIT criterion for alcohol problem—past year	32.0
Meets TCU criterion for drug problem—past year	32.0
Meets criteria for drug or alcohol problem—past year	44.0
**Sexual behavior and history**	
If male, non-heterosexual sexual orientation (bisexual, queer, other) and/or past sexual contact with men over the lifetime	30.0
Sex without a condom in the past month	40.0
More than one sexual partner past month	36.0
Lifetime exchange sex for money, drugs, or place to stay	48.0

Table 2 describes linkage to medical care and HIV outcomes, including use of ART and CD4 and VL results from the structured assessment battery, MRFs, and/or study blood draws. Most participants interviewed at the T2 assessment (*n* = 19) already had a regular health-care provider (84%) and usually received care in a hospital clinic (47%), other clinic (21%), or private doctor’s office (16%). Regarding the first follow-up period (T2/T3), MRFs and data from the assessment battery indicate 80% were linked to care within 90 days of diagnosis and/or by T2/T3. The mean CD4 count was 569 cells/mm^3^ (SD = 364 cells/mm^3^), based on MRF or blood draw, and the mean log_10_ transformed VL was 2.9 (SD = 1.4), by MRF or blood draw. About half (52%) had taken ART in that period; 82% of these participants had continued with ART. At the T4/T5 follow-up interview(s), 96% had seen a health-care provider for their HIV diagnosis. Most (96%) reported completing CD4 and VL testing at least once, with an average CD4 result of 559 cells/mm^3^ (SD = 357 cells/mm^3^) and an average log_10_ transformed VL of 2.4 (SD = 1.4 log_10_ transformed VL), based on MRF or blood draw. Approximately two-thirds (69%) were retained in HIV care over the previous 12-month period. At the first follow-up period, 39% had undetectable VL, and 62% had undetectable VL by the final follow-up period. Most had taken ART by the final follow-up point (87.0%). In 7 out of 16 MRFs, we found evidence of an HIV diagnosis prior to study involvement (44%; data not shown).

**Table 2:** **Patterns of linkage to HIV care and clinical outcomes**.

Medical care before T2 interview		
Has regular health-care provider (T2 only)	84.2% (16/19)	
**Type of facility you usually receive your medical care (T2 only)**		
Hospital clinic	47.4% (9/19)	
Clinic not based in a hospital	21.1% (4/19)	
Private doctor’s office	15.8% (3/19)	
Emergency room	10.5% (2/19)	
Community-based organization	5.3% (1/19)	

	**Time 2/3 Mean (SD)/%**	**Time 4/5 Mean (SD)/%**

**HIV primary care**		
Have you seen a health-care provider for your HIV diagnosis	78.3% (18/23)	95.7% (22/23)
Linked to care within 90 days of diagnosis [medical report form (MRF)]	81.3% (13/16)	
Health-care provider recommended antiretroviral therapy (ART)	66.7% (12/18)	91.3% (21/23)
Prescription filled	100% (10/10)	100% (19/19)
Retained in care over 12 months (MRF)	68.8% (11/16)	
**CD4**		
Had CD4 taken (interview)	94.4% (17/18)	95.5% (21/22)
CD4 (blood draw and/or MRF)	M = 569, SD = 364, *N* = 23	M = 559, SD = 357, *N* = 20
**Viral load (VL)**		
Had VL taken (interview)	94.4% (17/18)	95.5% (21/22)
VL self-report undetectable (interview)	54.5% (6/11)	83.3% (15/18)
log_10_ VL (blood draw and/or MRF)	M = 2.9, SD = 1.4, *N* = 23	M = 2.4, SD = 1.4, *N* = 21
Any VL < 50 (undetectable VL) (blood draw and/or MRF)	39.1% (9/23)	61.9% (13/21)
**ART**		
Ever took ART and/or took ART since last interview (interview)	52.2% (12/23)	87.0% (20/23)
Has continued with ART (interview)	81.8% (9/11)	95.0% (19/20)

### Summary of Quantitative Findings and Qualitative Research Questions

Overall, these quantitative data show good engagement in HIV care and clinical outcomes but suggest that a substantial minority (~20%) were slow to engage in HIV care, and most experienced delays in achieving good adherence to ART in order to achieve undetectable VL (~60%). Yet these gaps in HIV care engagement and ART uptake were reduced by the final follow-up point. Further, we found a substantial proportion of the cohort had been previously diagnosed with HIV, but had not elected to disclose their HIV status to the study at enrollment. Thus, guided by the theoretical model and the review of the literature described above, the answers to a number of main qualitative research questions seem relevant to understanding these results: how did participants experience and adapt to the receipt of the HIV diagnosis, whether it was new to them or not, and how did this experience influence their motivation and/or abilities to engage in HIV care and/or initiate ART? Embedded in this question is an exploration of the reasons why participants did not disclose their HIV status to the study at enrollment. In addition, what other factors promoted or impeded engagement in HIV care and uptake of ART with good adherence over the study period?

### Qualitative Results

#### Overview of Qualitative Findings

Drawing on both prospective and retrospective reports, we found acceptance of a new HIV diagnosis was often not a simple, binary phenomenon whereby participants thought of themselves as not being infected with HIV one moment, were tested and told of their positive test result, and then accepted that diagnosis as true or accurate in the next. Instead, we found the state of “knowing” one was infected with HIV at the time of receiving the new diagnosis, whether in the course of the present study or prior to it, was complex, multi-faceted, and evolving over time. With respect to prior knowledge of the HIV diagnosis provided in the study, some participants reported no previous knowledge of the diagnosis, and others reported having concerns and suspicions they were infected (e.g., because a past partner was infected). Some participants had been informed of an HIV diagnosis in the past but had not personally accepted that diagnosis as accurate, while a small number had been previously diagnosed and had integrated and accepted the diagnosis into their self-concept, which allowed for HIV-related risk reduction, disclosure, and health-care behaviors. Indeed, regardless of whether they had accepted their HIV status during the course of the study, we found it was common, if not typical, for participants who had been told in the past they were HIV infected to have found themselves unable to accept or integrate that knowledge into their sense of self, sometimes for as long as a decade or more. Yet even these participants, who could be described as “in denial” and whom we characterize as experiencing “delayed acceptance,” at the same time often evidenced some level of “knowing” they were HIV infected, as we describe below. Thus, we found some participants reported both knowing and not knowing about their HIV-infected status simultaneously.

Thus, we characterized the primary reactions to a new HIV diagnosis evident in the present study as falling into the following typologies: (1) there was no expectation of an HIV diagnosis, the diagnosis precipitated a crisis, and acceptance of the new diagnosis was slow; (2) there was suspicion of an HIV diagnosis, and the information was met with acceptance; (3) the new diagnosis revealed a combination of denial and some level of knowing about the diagnosis; and (4) the participant was, in fact, already aware of the past diagnosis, but chose not to disclose it to the study. Below, we present case studies that illuminate the main aspects of each of the first three typologies, including their implications for engagement in HIV care and uptake of ART, as well as the cases’ relationships to themes found more broadly in the qualitative analysis. For parsimony, we do not describe the fourth typology, but attend to this group in the Section “Discussion.”

The case studies presented below describe male participants, because almost all enrolled in the study were men. We attend to commonalities among men and women and themes specific to women in a brief section below. The names used below are pseudonyms, and identifying details have been changed to protect participants’ confidentiality. For context, the case studies include some description of participants’ experiences in the study’s first phase, when HIV testing was provided, as well as the second, which focused on providing post-test counseling and linkage to HIV care. Further, cases reflect participants’ natural histories, as well as their engagement with the research project, which provided navigation (intervention arm) or standard care (control arm).

#### Case A: Newly Diagnosed—Crisis and Slow Acceptance (Intervention Arm)

Jerry was a 34-year-old African-American/Black man in an on-again/off-again relationship with a woman who was the mother of the youngest of his three children. Jerry had been incarcerated for varying lengths of time in the past, and partly as a result of these incarcerations, he was unemployed and in need of stable housing when he entered the study, residing only temporarily with his mother.

Upon consenting to the rapid HIV test (i.e., OraQuick rapid HIV test with oral fluid) and receiving a preliminary positive test result, Jerry expressed what he later described as an almost overpowering sense of fear, shock, and disbelief, as well as an inability to accept the veracity of the test results. In an effort to facilitate his acceptance of the new diagnosis, he was offered the opportunity to view the actual HIV test device that indicated the positive result, but he declined. He was then asked if he would agree to participate in post-test counseling with the study interventionist, despite his inability to accept the HIV test result as accurate. While his first impulse was to flee from the room and leave the facility, with great reluctance he did elect to stay and discuss the test result. In keeping with the study protocol and ethos, his autonomy was respected as study activities were offered to him, and his choices were solicited and respected.

During the course of the post-test counseling session, the interventionist sought to help Jerry manage these powerful negative reactions to the diagnosis, prevent escalation to a larger crisis, and encourage Jerry to stay engaged with the research study. In fact, despite Jerry’s severe negative emotional reactions, he did agree to stay in touch with his interventionist and to ask questions and seek support from her and others as needed. At the end of the second intervention session (the last activity in the study’s first phase), Jerry was still unable to accept the HIV diagnosis as true or accurate. However, Jerry consented to a confirmatory HIV test (the laboratory-based OraSure test using oral fluids) and provided a blood specimen for CD4 and VL testing. He agreed to schedule his post-test counseling intervention session, which would take place 2 weeks later, where he would receive confirmatory HIV test results. During this interim period, the confirmatory test results were received, which indicated that consistent with the rapid HIV test results, Jerry was indeed infected with HIV.

Over the next 5 weeks, Jerry repeatedly canceled appointments for his post-test counseling intervention session, and reported to his interventionist he was not yet emotionally prepared to meet and discuss his HIV diagnosis. He was, in fact, still feeling overwhelmed by the prospect of an HIV diagnosis. As he later recalled,

HIV is another level. It’s not like gonorrhea, syphilis, chlamydia, crabs, it’s a whole ’nother level. You know what I’m saying? I was nervous.

Five weeks passed. Jerry was still not ready to receive his confirmatory test results from the research study. Yet, during that period, he stayed in touch with his study interventionist. He informed her he sought advice from his primary care physician and consented to a blood-based laboratory HIV test from him. However, Jerry delayed receiving the test results from the primary care provider as well, as he reported:

Like I said, I still wanna get the results from the blood first before everything. I really didn’t do nothing yet about [confirming my diagnosis]. Like I said, I’m scared, but at the end of the day I’m still gonna have to—I have to accept it and just go up in there [to the primary care provider] and see what’s goin’ on. But, I ain’t really doin’ it yet.

Consistent with the motivational interviewing approach, the study interventionist did not pressure him to accept the diagnosis, but instead encouraged him to explore his personal reactions to the test results she had provided, make decisions about the steps he would take to regain emotional equilibrium, evaluate his sexual behavior in light of this information, and seek out support.

Especially during the times when Jerry wished to disavow the positive test results he received from the research study, he would return to the hope that the results were inaccurate because he received a test using oral fluids during the research study, but not a blood specimen. In fact, he cited this as a reason for the delays he experienced accepting the truth of his diagnosis:

I put it like this, I’m not saying nothing to no one [about the diagnosis] until I take that blood test. ’Cause even though by swab, it could be in your saliva, it doesn’t mean it’s in your system. So, when I go do that blood work, and I find out the real results, then I could be able to open up about it. Right now, I don’t wanna say nothing ’bout it.

Yet Jerry also continued to express apprehension regarding receiving his HIV blood tests results from his primary care provider. He acknowledged that he was waiting to feel “ready” to receive these test results before acknowledging to himself and others that he was infected with HIV, and then seeking HIV care. Jerry understood that eventually he would need to do so. But, he would do so in his own time, as he explained:

Cause I don’t wanna know the full answer … I’m saying I’ll go do it, I just wanna take it one step at a time. You know what I’m sayin’? I’ll do it by myself, but I’m just not ready right now. But Imma go. Eventually, Imma go.

Furthermore, in addition to reporting anxiety around the potential “*death sentence*” that Jerry associated with HIV infection, he also noted fears of stigma surrounding being infected with HIV. For example, although Jerry was in need of housing, and the local social services administration provides housing for people living with HIV, Jerry’s fear of HIV-related stigma prevented him from accessing those services, as he recalled:

I don’t wanna go to no HIV housing. ’Cause once you go to HIV housing, everyone already knows you—what it is.

Despite not accepting or, at least, not fully accepting the diagnosis, Jerry began to evaluate his sexual behavior. Indeed, Jerry had an active sex life with a substantial number of different partners. Jerry expressed concern about how HIV might negatively affect this important aspect of his life. In particular, Jerry was concerned about the effectiveness of condoms and how to negotiate safer sex. He reported a lack of confidence about his understanding of HIV infection and how HIV is transmitted to partners. Again, although the study interventionist was, in theory, able to refer him to sexual health services to bolster his personal secondary prevention efforts, his difficulty accepting the HIV diagnosis limited his willingness to do so. Yet he continued to engage with his study interventionist by phone, and consider changes he might need to make–if and when he was ready to accept the diagnosis.

Throughout his time enrolled in the study, Jerry continued to struggle to accept that he was, in fact, infected with HIV. Jerry did not agree to receive confirmatory results of his HIV test from the study or participate in the post-test counseling intervention session, although, notably, he did return phone calls from the interventionist, engage in discussions with her, and agree to participate in other project activities including the in-depth qualitative interview, blood draws, and a structured assessment battery.

**Figure 2:**
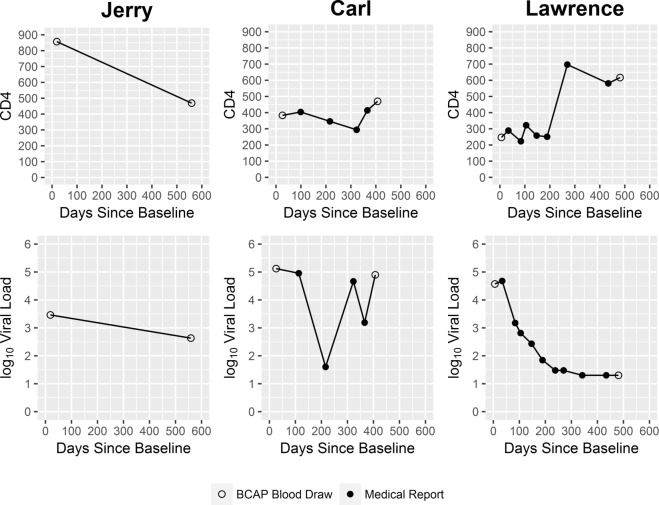
**Clinical outcomes for case study participants**.

At the time he concluded his participation in the study, Jerry evidenced only very modest progress in his abilities to accept and adapt to his new diagnosis. Because his fears and anxieties surrounding the meaning of an HIV-positive status seemed insurmountable to him, he could not bring himself to receive either his confirmatory test results from the research study or his blood test results from his primary care provider. Given that he could not accept that he was HIV infected, he did not seek HIV primary care. HIV biomarker data collected from him at two time points over the course of the study (Figure 2) showed decline in his CD4 count. His VL also decreased in this same period. Taken together, these CD4 and VL data suggest Jerry was recently infected with HIV at the time he entered the research study, and coming to the end of the acute infection stage, when VL levels are initially very high but then decrease, and HIV antibodies develop (56).

In the sample more generally, we found, in both retrospective and prospective reports, slow acceptance of a new HIV diagnosis was common in this cohort. Further, Jerry’s case highlighted a number of themes found more broadly in this study; for example, that HIV may be considered a “death sentence,” even in the context of current highly tolerable and effective ART regimens. Further, participants commonly reported they had insufficient knowledge surrounding HIV transmission and HIV prevention. Finally, fears regarding the ways in which an HIV diagnosis might interfere with or even foreclose upon future intimate relationships, the importance of maintaining sexual and romantic relationships, and the critical role of stigma, were potent barriers to acceptance of HIV status. Certainly, we found that learning one has a life changing and stigmatized diagnosis does not always result in timely acceptance of that knowledge, and a lack of acceptance can be a powerful barrier to the receipt of HIV care.

#### Case B: Newly Diagnosed—Met with Acceptance (Intervention Arm)

Carl was a 58-year-old African-American/Black man staying in a homeless shelter at the time he enrolled in the study, where, instead of a bed, he slept upright in a chair because of overcrowding. Carl reported he had not been engaged in any type of medical care for as long as he could remember, only visiting an emergency department if it was unavoidable. Further, he indicated he suffered from some cognitive impairment due to an earlier traumatic brain injury, making it difficult for him, for example, to plan or enact sequences of new activities. Moreover, at the time he entered the study, Carl felt run-down and sick. He had trouble walking and had been drinking heavily over the last several months. Carl reported he had never been tested for HIV, but that he suspected that he might be HIV infected. He noted that after the death of his wife 15 years prior, he had started to drink daily and engage in what he considered risky sexual behaviors with a number of partners. When Carl was offered an HIV test in the study’s first phase, he began to prepare himself emotionally for the possibility of receiving an HIV diagnosis.

After receiving his preliminary positive HIV test results from the rapid test, Carl was upset, but remained calm and maintained perspective. He later recalled,

It really hurt when I found out I had it. It was a big blow. But I’m gonna take one step at a time. It’s a long, long road.

Immediately after learning his status, Carl stated he was willing to obtain HIV primary care, but explained that he was unable to do so because he had no phone and no means of transportation—common barriers to HIV care among this population. After providing him with the confirmatory test results and post-test counseling in session 3, he was also given a phone and funds for round-trip local public transportation—simple and practical, yet critical, forms of assistance.

Moreover, cognitive challenges related to the traumatic brain injury compounded the difficulties Carl faced linking to HIV care and other social services. Yet, with concrete guidance from his study interventionist, including assistance with paperwork to apply for public insurance (Medicaid), Carl was referred by the study to a comprehensive care social service agency, which provided case management, nutrition support, support groups, housing assistance, and HIV primary care—a setting that seemed optimal given Carl’s needs.

Soon after, Carl attended his initial HIV primary care appointment at this multi-service organization. Much to his surprise, in addition to HIV infection, he was also diagnosed with diabetes. He then initiated ART and treatment for diabetes and reported being pleased with a rapid and dramatic improvement in his health. He noted, “*I always take my medication. I feel healthy [now], like I was 17*.”

However, at times it was challenging for Carl to adhere to his HIV medications. For example, at one point he took a trip to visit family who did not know about his HIV status. Because Carl was not yet prepared to disclose his HIV status to these family members, he left his HIV medication at home rather than risk his family members’ accidently learning of his HIV status. Consequently, Carl’s VL, which had dropped dramatically in response to the ART regimen, rebounded, as shown in Figure 2.

Periodic contacts during the navigation period were used to help him identify barriers to maintaining good adherence to ART and making good decisions about his health behaviors. Upon learning of the effect his non-adherence to the ART regimen had on his VL levels, Carl was upset and re-committed to adhering well to the medication. Further, Carl enrolled in a conditional economic incentive program at the organization; that is, an intervention that reinforces adherence to HIV medication regimens with economic incentives provided when participants achieve undetectable VL. Carl found the program beneficial, as he stated:

(I) just take my medicine, take one pill. When I miss a lot of days … [my viral load] goes back up. My [viral load] when I first went in, was like, over 100,000. Now I got it down low. I think it’s down to 22,000. But I got to get it back to 40 (undetectable). Because, see once I get it to 40 again, I get $150 bonus card.

Importantly, over the course of his participation in the study, Carl developed positive relationships with his doctor and case manager, and reported having “*complete faith*” in the information they provided to him regarding management of his HIV infection. Further, Carl moved from the shelter into an apartment with a life-long friend. In navigation contacts, he weighed the pros and cons of sharing his HIV status with this person and decided to do so. In fact, this friend was an ongoing source of support and was the only person to whom Carl disclosed his status during the study. Further, through the social service organization where he received services and HIV care, Carl applied for supplemental security income and housing for HIV-infected persons. Unfortunately, though, before his city-sponsored HIV housing came through, Carl had to leave his friend’s apartment. He acknowledged this was stressful for him, saying, “*I lost weight. Probably stress. Because I got to go back to the shelter*.” Carl asserted, however, that he was able to maintain his health and good ART adherence while waiting for housing, even if he had to live in a shelter, noting, “*no, I’ll take my medication with me. I still see my doctor*.”

Carl stated he was motivated to reduce his chances of transmitting HIV to others by taking ART, and by refraining from sexual behavior at this point. He was still considering how he might negotiate condom use and disclosure to sexual partners in the future, as he stated, “*Because I wouldn’t give them the HIV. You don’t want nobody to give you that, right?*”

Over the course of the study, Carl’s physical health showed improvement. His CD4 increased. Yet consistent with his reports of inconsistent adherence, his VL data were variable (Figure 2).

Carl’s case highlights a number of themes common among participants receiving a new diagnosis. First, while Carl may have been concerned he was infected with HIV, he had never been tested for HIV. He was brought to the study through active outreach from a peer as part of RDS and agreed to be tested. With respect to the peer recruitment component of the study he noted,

No, I didn’t know [I was infected with HIV]. I really didn’t. So, in a way that guy like saved my life when he give me the card to come here.

Further, like many participants in the study, Carl was faced with a number of potent structural barriers to his timely engagement in HIV medical care, including lack of transportation and difficulties applying for public health insurance. There is growing awareness in HIV testing and clinical settings regarding the need to resolve or circumvent such barriers to health care, similar to the approach taken in the present study. Once linked to a multi-service organization, Carl developed an open and trusting relationship with his health-care and social service providers. This setting provided him with high-quality HIV care and assisted him with managing his other significant life stressors, in particular his diabetes and housing instability. Moreover, similar to Jerry’s experiences, and results in the sample more broadly, fear of stigma was a primary theme. Finally, Carl lacked confidence in his ability to negotiate safer sex and struggled to find a way to have a satisfactory sexual and romantic life while living with HIV.

#### Case C: Difficulty Accepting HIV Status over a Long Period of Time (Control Arm)

Lawrence, a 42-year-old African-American/Black man, entered the project in the midst of a marital crisis. Lawrence and his wife, Brenda, had been married for 10 years and had three children together. He was employed by the local transit authority, while she stayed home with the children. Lawrence was recruited into the study by Brenda, who, 7 months pregnant with their fourth child, tested positive for HIV during the course of her own participation in the first phase of this research study. Although Brenda had been tested for HIV early in her pregnancy and found to be uninfected, she had seroconverted by the time she reached her seventh month.

Prior to her being tested in the study, Brenda had asked Lawrence to participate in the research project, as part of her peer recruitment activities. However, Lawrence was non-committal and put her off. But after Brenda was found infected with HIV, she again asked Lawrence to present to the study. Under these circumstances, Lawrence agreed to enroll. He participated in HIV counseling and testing and received confirmation that he also was living with HIV.

Lawrence’s initial reactions were guilt and concern for his wife and children. In fact, he was not surprised by Brenda’s test results. He reported, with hesitation and distress, that over a decade earlier he had a former female partner who he “*vaguely remembered*” was found HIV infected after they had ended their relationship. She contacted him at the time she was diagnosed, and he was tested soon after. Lawrence reported he found it difficult to remember the details of this period of his life with any specificity, but recalled he was told at this time was HIV infected. But, as he went on to explain, he did not tell anyone of his status, and did not engage in HIV care. Lawrence could not bring himself to fully accept the diagnosis. He reported on the one hand, he “knew” he had HIV because he had been told by a medical provider he was HIV infected. At the same time, for reasons he had difficulty articulating, he could not allow himself to know that he was HIV infected.

Lawrence kept his HIV diagnosis largely, but not entirely, out of his own awareness over the next 10 years, as well as he could, in part by drawing on a misconception that if someone had no discernable symptoms of HIV, there was no need for concern regarding HIV’s impact on the body. Lawrence believed his HIV infection existed in a “cocoon” in his body, which would only affect him when it “burst,” which he believed only took place around the time that Brenda became infected with HIV. As he recounted:

Like I said, I’m lucky because how I got contacted with [HIV] was like about 10 years ago, after the girl I was with [and before] I met my wife. But before I met [my wife] I got hit, you know what I mean? But it somehow, some way it was in a cocoon or something like that, or whatever in my body. It recently burst. Now if it had been opened [before], God only knows how bad it would have really gotten.

Lawrence relied on the fact that Brenda had tested HIV negative during her previous pregnancies as another rationale for not disclosing his HIV status to her or integrating the diagnosis into his sense of self. In other words, as time went on, and Brenda remained uninfected with HIV, he became increasingly convinced that he would not transmit HIV to her. Yet, a sense of guilt ate at him, and he lived in fear she would find out about his HIV status and leave him. This fear of the marriage ending appeared to be the primary reason Lawrence did not disclose his HIV status to her, or fully acknowledge it to himself.

Thus at the time he was diagnosed, Lawrence’s primary concerns were regarding his marriage, as he was certain Brenda would leave him once their HIV statuses were revealed. As he recalled,

But her, it counted more, you know what I am saying, because she got [it] through me, so I thought the worst, I thought she was gonna leave me, take the kids and all that … I was shocked, because I thought she wanted to kill me. You know what I am saying, I thought she wanted to kill me at first, I was like, oh, can I sleep with you, can I live in this house—can I stay in here?

Yet Lawrence’s fears did not become reality. Brenda and Lawrence did not end their marriage, and in fact relied on one another as they weathered one health decision after another. Brenda received timely medical intervention to prevent mother-to-child transmission of HIV to their child, gave birth by Cesarean section, and stayed on ART after the pregnancy. Because the diagnosis was made late in the pregnancy, the baby was put on ART after birth for a period of time, and Brenda could not breastfeed, which caused her sadness. However, the baby was eventually pronounced free of HIV infection. One by one, the other children received HIV testing, and all were found uninfected.

Lawrence reported being highly motivated to engage in HIV care on a consistent schedule, initiate ART, and adhere to his ART regimen, in part in response to the guilt he felt over how things transpired, and due to his newfound perspective on his HIV infection. In fact, he described his HIV diagnosis as a life-changing experience and suggested he felt relief at not having to hide his status from himself and others. Based on HIV biomarker data collected over the course of his enrollment in the study, Lawrence’s physical health showed marked improvement: his VL decreased over his time in the study, and his CD4 cell count increased (Figure 2). The study’s motivational interviewing approach and non-judgmental ethos, which Lawrence described as “*open-minded*,” “*like a family*,” “*giving (him) hope*,” and “*caring*,” played an important role in helping Lawrence manage this painful, critical period in his life.

Lawrence’s fears surrounding the potentially negative effects of an HIV disclosure on his intimate relationships served as an impediment to his accepting his own HIV diagnosis over a long period of time, with significant costs to his sense of self and well-being. As noted above, a long and difficult trajectory to accepting one’s diagnosis was common in this sample. Thus, Lawrence’s case highlights how accepting an HIV diagnosis can be a lengthy and complex process for many PLWH, sometimes with serious negative consequences for one’s health and the health of loved ones. Further, low HIV health literacy, such as the idea that HIV lives in a cocoon, may impede acceptance of a new HIV diagnosis, as many PLWH who feel healthy express misconceptions about HIV, such as a lack of a need for treatment until one is ill.

#### Themes Specific to Women

The analyses presented above focused primarily on the experiences of men, given the nature of the data set, and congruent with local epidemiology where more men than women are diagnosed with HIV. Nonetheless, we found a number of results specific to high-risk heterosexual women.

As we described above, men commonly feared the loss of their sexuality and sexual relationships, and this caused distress and impeded their accepting their diagnoses. Yet, interestingly, this theme did not arise among female participants. Instead, women commonly described their responsibilities caring for children and family members as primary motivators for self-care. Moreover, they described a number of challenges related to disclosing their HIV status to their children, such as if, when, and how to disclose. Men, on the other hand, did not reference either caregiving or children as aspects of their adaptation to HIV. For example, as Jonelle, a 57-year-old African-American/Black woman described,

When I found out [I had HIV], I was in denial for a long time. But I had to grasp myself because I got children …. I went down to 90 pound with bricks in my pocket. And then, I got tired of my kids worrying about me. All the nurses and everybody kept calling, and I got tired of the way I looked at myself. I really looked in the mirror at myself and I thought, damn [Jonelle], you like 55-years old, you look like you’re 90-years old. And I just had the crap scared out of me, scared right? That’s all, I had to get right.

Finally, the theme of betrayal arose for both sexes. However, women more commonly than men described being “betrayed” by male partners who transmitted HIV to them, either unknowingly, or in some cases, and consistent with the cases described above, in the context of the male partner’s knowledge, at least on some level, that he was infected with HIV. As Jonelle described, “*I found out that person that I was with had HIV. And he was sayin’ that he didn’t know that he had it. He told me he didn’t know and then, when the red flag went up [and I was suspicious about his status], he knew. He knew*.”

## Discussion

Among high-risk heterosexuals, reactions to receiving a new HIV diagnosis are complex and multi-faceted. We found difficulty accepting and internalizing one’s HIV status is frequently a challenge and is often a central reason for non-disclosure of HIV status to friends, family, sexual partners, and even researchers. Indeed, drawing on both prospective and retrospective qualitative data, we found knowledge of one’s HIV status in this population is not typically a simple binary phenomenon after diagnosis, even for months or years after diagnosis. That is, participants typically do not think of themselves as uninfected with HIV one moment, then after being informed the HIV test is positive, immediately understand, accept, and/or “know” that they are infected. Rather, we identified four main ways in which high-risk heterosexuals react to receiving an HIV diagnosis (namely, there was no expectation of an HIV diagnosis, the diagnosis precipitated a crisis, and acceptance of the new diagnosis was slow; there was suspicion of an HIV diagnosis, and the information was met with acceptance; the new diagnosis revealed a combination of denial and some level of knowing about the diagnosis; and the participant was, in fact, already aware of the past diagnosis, but chose not to disclose it to the study). These reactions, for the most part, can be placed on a continuum of acceptance of HIV status, ranging from no acceptance at all (i.e., denial or delayed acceptance) to full knowledge and acceptance of HIV infection. Moreover, years might pass while individuals struggle to become ready to accept an HIV diagnosis. Yet not accepting one’s HIV diagnosis has serious potential adverse public health consequences, because such acceptance is typically a prerequisite for engagement in HIV care, uptake of ART, disclosure to others, and minimizing the risk of transmission to sexual and injection drug using partners.

The sequential explanatory mixed-methods design is useful in describing and uncovering the complexities that underlie reactions to a new HIV diagnosis in a vulnerable population; data sources, while addressing different research questions, shed light on complementary aspects of the phenomena under study. Thus, the present study advances research on adaptation to a new HIV diagnosis in a vulnerable population by uncovering the complexities high-risk heterosexuals experience in the process of acceptance, as well as some of the specific barriers to acceptance, most notably fear of stigma and of the potential loss of vital intimate relationships. Interestingly, Horter and colleagues (57) found markedly similar results in another high-risk context, Swaziland. They found the process of acceptance of HIV diagnosis is non-linear and varies temporally, with some individuals experiencing non-acceptance for extended periods of time. Further, consistent with the present study, acceptance of HIV status was a necessary precursor to engagement in HIV care.

Moitra and colleagues (58) have developed an acceptance-based behavior therapy intervention for newly diagnosed PLWH to increase acceptance, reduce perceptions of HIV stigmatization, and increase disclosure of HIV status to social supports (58). Our research highlights the utility of such an approach, not only for those newly diagnosed but also for PLWH more generally who evidence slow progress toward acceptance of HIV diagnosis. Further, findings from the present study indicate that for interventions to foster acceptance of HIV status among the population of high-risk heterosexuals, cultural tailoring will likely need to include attention to the meaning and real-life implications of stigma, as well as potential adverse effects of the diagnosis on romantic and sexual relationships. For example, Bowleg and colleagues (59) have found that for African-American/Black men, a number of explicit ideologies of masculinity operate (i.e., what it means to be a “real man”) that may complicate acceptance of HIV status in the context of one’s sexual relationships. In particular, they highlight norms that Black men should have sex with multiple women, often concurrently; they should not decline sex—even risky sex; and women should be responsible for condom use (59). Thus, the psychosocial losses associated with an HIV diagnosis among high-risk heterosexuals are great, including the potential negative impact on the sense of self; and these losses appear to impede acceptance of one’s HIV status. Women are also concerned about the effects of HIV on their relationships, but in contrast to men, focus not on sexual and romantic relationships, but on the challenge of disclosure of their HIV status to their children and other family members. Yet relationships with these family members also prompt women to face their diagnoses and engage in HIV care. In light of the small numbers of women in the present study, more research on adaptation to HIV diagnosis among women is needed, as noted below in the Section “Limitations.”

### Patterns of Engagement in HIV Care and ART Uptake

We found a substantial proportion of participants, at least 44%, were not newly diagnosed in the course of the study, and some of these with past HIV diagnoses had already engaged in HIV care, although they did not elect to disclose their HIV status at the time they enrolled. As Marzinke and colleagues (60) have described, other similar studies to identify undiagnosed HIV infection also found substantial rates of non-disclosure of HIV status, particularly when participants would be denied financial compensation if they so disclose. The present study was designed to increase the proportion of participants who disclosed past HIV diagnoses at study enrollment by providing comparable activities and compensation levels for participants who were HIV infected and those whose HIV status was uninfected or unknown. Yet despite the fact that a substantial proportion of the “newly diagnosed” sample was, in fact, previously diagnosed with HIV, rates of viral suppression were poor at the first follow-up point, suggesting slow uptake of ART, as we discuss below. Taken together with the qualitative findings, the present study provides critical insights into some of the reasons for delayed engagement in HIV care and uptake of ART and the role of acceptance of one’s HIV status as a vital facilitator of care engagement.

In the sections that follow, we refer to participants in the present study as newly diagnosed, but with the understanding that a substantial proportion had at least some level of previous knowledge of their HIV infection, and some were already in HIV care. Nonetheless, they were “newly diagnosed” in that they presented to the larger study with self-reported HIV-uninfected/unknown status and were informed over the course of the study they were infected with HIV.

Participants in the present study were primarily male and African-American/Black. Consistent with the understanding that HIV is a disease of low socioeconomic status and structural inequality (7), they evidenced a wide range of serious risk factors, including poverty, substance use problems, homelessness, unemployment, and past incarceration. Yet most had health insurance and/or regular health-care providers at the time of diagnosis. We found successes in HIV care linkage and retention post-diagnosis, as well as gaps. Most (approximately 80%) were linked to HIV care within 90 days of diagnosis, as recommended by the CDC (5), and almost all (>95%) had seen a health-care provider for HIV infection by the final follow-up period. We speculate participants’ pre-existing relationships with health-care providers, in conjunction with the study intervention components, fostered subsequent linkage to HIV care over the follow-up period, even in the context of serious risk factors that typically serve as barriers to primary care (61). Yet past research has shown that African-American/Black men overall are less likely than their White peers to present for primary care on a regular basis (62), suggesting that boosting rates of regular primary care use in this population can serve the goal of fostering timely engagement in HIV care among those later found to be HIV infected.

Overall, the cohort evidenced satisfactory rates of engagement in HIV care and uptake of ART by the final follow-up period, and clinical indicators were generally stable. Approximately two-thirds (68.8%) were adequately retained in HIV care over the nearly 1 year post-diagnosis follow-up period, based on reports from the medical record. These rates are higher than found nationally (63), but a third evidenced insufficient engagement, suggesting that interventions of a longer duration or greater intensity are needed. Moreover, consistent with findings from the present study, Rajabiun and colleagues (64) found that participants’ levels of acceptance of their HIV status was associated with cycling in and out of care. With respect to gaps in engagement along the HIV care continuum, approximately 20% had not engaged in HIV care, almost half had not initiated ART, and nearly 60% had not achieved VL suppression at any point during the first follow-up period. By the final follow-up period, almost all had engaged in HIV care (>95%), but 13% had not initiated ART, and almost 40% had not achieved undetectable VL during this period. Yet leaders in the field have noted the objective of elimination of HIV transmission depends on the 90–90–90 goal; that is, 90% of PLWH being diagnosed, 90% of these being on ART, and 90% of these being virally suppressed (65, 66).

Although we cannot say definitively due to a lack of a control group and small sample size, these generally positive clinical outcomes point to the potential importance of integrated culturally appropriate seek, test, treat, and retain approaches, such as the program described in the present paper. Further, these findings underscore the potential utility of providing ongoing linkage services in HIV testing sites to bring newly diagnosed individuals to HIV care and provide support during the transition to care, which can be lengthy, particularly for those with multiple barriers to accessing medical services (67, 68).

### Staying Engaged with Participants in Times of Crisis

The qualitative study component findings highlight the success of the study’s intervention components in engaging and retaining highly vulnerable participants through times of crisis. In particular, the intervention’s ethos of non-judgment, anti-stigma, and acceptance, grounded in motivational interviewing (36), was critical to maintaining close contact with participants through difficult circumstances, such as those described in the case studies. Findings also underscore the complexity of HIV testing programs using peer-to-peer recruitment methods, where sero-discordant sexual partners, injection drug using partners, and friends and family members may enroll in the study. To manage participant confidentiality and clinical needs it may be necessary to employ highly skilled clinicians with an understanding of the full range of potential reactions to a new HIV diagnosis, as described above.

### Limitations

Study limitations include the small sample size of newly diagnosed individuals in comparison to estimates derived at the time the study was planned (25). This small sample size precluded the examination of the efficacy of the peer-driven intervention in the second phase of the study and also likely reduced the precision of some estimates. Further, there were more newly diagnosed individuals in the control than intervention arm, but the small sample size does not allow us to examine whether this is a meaningful difference. Further, as noted above, while we could estimate the minimum proportion of participants with past HIV diagnoses, it was not possible to determine this rate with precision. It was challenging to obtain MRFs in some cases because health-care settings, providers, or participants were not responsive; this resulted in some missing data. Nonetheless, retention to all other activities, including blood draws conducted by the study, was high (>80%). MRFs, therefore, may not be the optimal means of data collection for this population. Moreover, the qualitative data analyses did not yield many themes specific to women, and the three optimal case studies selected were of males. Future research on adaptation to HIV diagnoses among women is warranted. Finally, social desirability and other biases may have affected the accuracy of qualitative and quantitative data, although the triangulation of these data with each other and with medical record and laboratory reports may have improved validity of study findings.

## Conclusion

The present study extends the literature on the experience of receiving a new HIV diagnosis, focusing on a population at high-risk, and highlighting the complex and often-lengthy processes involved in accepting and adapting to a new HIV diagnosis. Yet, as we found, such acceptance is a necessary step toward engaging in HIV care and reducing sexual and drug use behaviors that might transmit HIV to others. Thus, attention to these processes in clinical settings has potential to identify PLWH with barriers to acceptance, address these psychosocial issues, and thereby improve HIV-related outcomes. Finally, the present study highlights the utility of the mixed-methods approach for complex phenomena such as reactions to an HIV diagnosis.

## Ethics Statement

The study was approved by the New York University School of Medicine Institutional Review Board.

## Author Contributions

AK directed the study, developed study procedures, developed the research questions, and conducted data analyses and writing. MG conceived of the overall study concept and design and played a primary role in writing the manuscript. CC participated in the design of the study, planned the statistical analyses, and helped draft the manuscript. NL participated in the design of the study and study implementation procedures, and assisted with conceptualizing and writing the manuscript. RF and AR analyzed qualitative data. TM-G, KH, and BM developed study procedures and participated in interpretation of data. All the authors read and approved the final manuscript.

## Conflict of Interest Statement

The authors declare that the research was conducted in the absence of any commercial or financial relationships that could be construed as a potential conflict of interest.
